# Clinical Application of Liver Imaging Reporting and Data System for Characterizing Liver Neoplasms: A Meta-Analysis

**DOI:** 10.3390/diagnostics11020323

**Published:** 2021-02-17

**Authors:** Lingling Li, Yixin Hu, Jing Han, Qing Li, Chuan Peng, Jianhua Zhou

**Affiliations:** Department of Ultrasound, Sun Yat-Sen University Cancer Center, State Key Laboratory of Oncology in South China, Collaborative Innovation Center for Cancer Medicine, 651 Dongfeng Road East, Guangzhou 510060, China; lill@sysucc.org.cn (L.L.); huyx@sysucc.org.cn (Y.H.); hanjing@sysucc.org.cn (J.H.); liqing1@sysucc.org.cn (Q.L.); pengchuan@sysucc.org.cn (C.P.)

**Keywords:** hepatocellular carcinoma, liver neoplasms, contrast media, ultrasonography, routine diagnostic tests

## Abstract

The Liver Imaging Reporting and Data System (LI-RADS) is a comprehensive system for standardizing liver imaging in patients at risk of developing hepatocellular carcinoma (HCC). We aimed to determine the diagnostic performance of LI-RADS category 5 (LR5) for diagnosing HCC and LI-RADS category M (LRM) for characterizing other non-HCC malignancies (OM) using contrast-enhanced ultrasound (CEUS) and computed tomography (CT)/magnetic resonance imaging (MRI). Multiple databases were searched for articles evaluating the diagnostic accuracy of CEUS LI-RADS and/or CT/MRI LI-RADS. A random-effects model was adopted to synthesize the summary estimates of the diagnostic accuracy of LR5 for diagnosing HCC and LRM for characterizing OM using CEUS and CT/MRI. The pooled sensitivity and specificity of CEUS LR5 for the diagnosis of HCC were 69% and 93%, respectively. The pooled sensitivity was 67% and the specificity, 93% of CT/MRI LR5 for HCC diagnosis. There was no significant difference between the overall diagnostic accuracy for HCC diagnosis of CEUS LR5 and that of CT/MRI LR5 in terms of diagnostic odds ratio (DOR) (*p* = 0.55). The sensitivity was 84% with a specificity of 90% in the CEUS LRM for characterizing OM, while the sensitivity and specificity of CT/MRI LRM for characterizing OM was 63% and 95%. The DOR of CEUS LRM for characterizing OM was higher than that of CT/MRI LRM without significant difference (50.59 vs. 36.06, *p* = 0.34). This meta-analysis indicated that CEUS LI-RADS is qualified to characterize HCC and OM and may provide complementary information on liver nodules to CT/MRI LI-RADS.

## 1. Introduction

Hepatocellular carcinoma (HCC) is a major cause of morbidity and mortality of cancer worldwide [1]. Unlike other malignancies, HCC can be non-invasively diagnosed in the absence of histological assessment. Imaging has been recommended as a vital diagnostic tool for HCC diagnosis in patients at high risk for developing HCC [2]. Hence, dependable imaging is critical.

CT and MRI are recommended as the first-line diagnosis methods for HCC diagnosis because of their powerful differential diagnosis of liver neoplasms [2]. In order to standardize reporting and data collection of CT and MRI for HCC diagnosis, the American College of Radiology (ACR) released the CT/MRI Liver Imaging Reporting and Data System (LI-RADS) in 2011 [3]. The three latest versions of CT/MRI LI-RADS were released in 2014, 2017, and 2018. An updated version in 2018 was finally adopted by the American Association for the Study of Liver Disease (AASLD) [4].

Along with the development of sonicated microbubbles, contrast-enhanced ultrasound (CEUS) is able to characterize focal liver lesions commendably. However, several studies have suggested that HCC could be hardly distinguished from some other non-HCC malignancies (OM), such as intrahepatic cholangiocarcinoma (ICC) and mixed hepatocellular and cholangiocarcinoma (HCC-CCA) in CEUS, thus leading to inappropriate clinical strategy [5,6]. That might be the main reason why the AASLD does not accept CEUS as a reliable diagnostic technique. However, other studies have found that when considering the arterial phase hyper-enhancement pattern and the onset and intensity of washout, CEUS did well on distinguishing HCC from OM [7,8]. In recognition of the usefulness of CEUS, several guidelines recommended it as the first- or second-line tool [9,10,11,12]. The CEUS LI-RADS was first introduced in 2016 for trial implementation and it was officially issued in 2017 [13]. Similar to CT/MRI LI-RADS algorithm, liver lesions are assigned into several categories ranked from LI-RADS category 1 (LR1, definitely benign) to LI-RADS category 5 (LR5, definitely HCC) with added categories LI-RADS category M (LRM, probably or definitely malignant but not HCC specific), LI-RADS category TIV (LRTIV, definite tumor in vein) and LI-RADS category NC (LRNC, cannot be categorized due to image degradation) in CEUS LI-RADS. Among them, LR5 lesions can be treated as HCC without biopsy, while LRM lesions need further confirmation through pathological assessment.

A recent systematic review explored the pooled proportion of HCC and overall malignancy in each LR based on CT and MRI [14]. Another two meta-analyses determined the sensitivity and specificity of LR5 for diagnosing HCC using CT/MRI LI-RADS and CEUS LI-RADS, respectively [15,16]. Kim et al. focused on the probability of HCC and OM in the LRM based on MRI [17]. Nevertheless, no systematic review or meta-analysis has compared the diagnostic performance of CEUS LI-RADS and CT/MRI LI-RADS for characterizing HCC and OM. Hence, we performed a meta-analysis to evaluate the diagnostic accuracy of CEUS LR5 for diagnosing HCC and CEUS LRM for characterizing OM in patients at risk of HCC in comparison to CT/MRI ones.

## 2. Materials and Methods

This meta-analysis was in line with the Preferred Reporting Items for Systematic Reviews and Meta-analysis statement [18]. The study protocol was registered in the INPLASY international platform of registered systematic review and meta-analysis protocols. The registration number was INPLASY202060056.

### 2.1. Literature Search

We systematically searched Ovid MEDLINE, Ovid Cochrane Central Register of Controlled Trials, EMBASE, and Scopus databases for studies published from 1 January 2014 to 7 April 2020 regardless of languages. The search strategy is shown thoroughly in Supplementary Table S1. The reference lists of the eligibility studies and meta-analyses related to the topic were also manually searched.

### 2.2. Eligibility Criteria 

The original studies that evaluated the diagnostic performance of CEUS LI-RADS or CT/MRI LI-RADS for characterizing HCC and OM in patients at high risk of HCC were included. Here, according to LI-RADS, adults who had cirrhosis, chronic HBV infection, and current or prior HCC were defined as patients at high risk of HCC. The exclusion criteria were as follows: (a) studies that did not enroll either HCC or OM, (b) studies in which the index test did not meet a minimum requirement of LI-RADS technical recommendations, (c) studies without an available reference standard, (d) studies with duplicated patients and (e) reviews, meta-analyses, case reports, letters, and comments. 

### 2.3. Study Selection and Data Extraction 

After removing duplicate records, two reviewers independently screened the titles and abstracts of the retrieved studies to remove the irrelevant ones. Full texts of the included abstracts were subsequently reviewed. Data of the eligibility studies were extracted by two reviewers independently using a pre-designed data form, shown in Supplementary Table S2. In cases of discordance, a third reviewer would discuss with the two reviewers until an agreement was reached.

### 2.4. Quality Assessment

Two reviewers performed methodological quality assessments independently using the Quality Assessment of Diagnostic Accuracy Studies 2 (QUADAS-2) tool [19] that contains 4 domains including patient and lesion selection, index test, reference standard, and flow and timing. A third reviewer would ask the two reviewers to state their reasons and made final decisions once inconsistency occurred.

### 2.5. Statistical Analysis

The pooled sensitivity, specificity with their 95% CI of LR5 for diagnosing HCC and LRM for diagnosing OM were calculated using a bivariate random-effects model. Diagnostic odds ratios (DORs) with their 95% CI were also calculated, which represented the overall diagnostic accuracy. It was worth mentioning that when more than one dataset were available in a study (e.g., >1 LI-RADS version, >1 index test >1 reviewer), the data were averaged. The Cochran Q test and *I*^2^ statistic were used to assess the heterogeneity. *p* < 0.05 of Q test and *I*^2^ > 50% may suggest apparent heterogeneity. Meta-regression analysis was performed to explore the potential source of the heterogeneity. Publication bias was investigated by Deek’s funnel plot and the statistical significance was tested using Egger’s regression test. All statistical analyses were performed with Stata version 13.0 (StataCorp, College Station, TX, USA) and Review Manager version 5.3 (Nordic Cochrane Centre, Cochrane Collaboration, Copenhagen, Denmark).

## 3. Results

### 3.1. Literature Search

**Figure 1:**
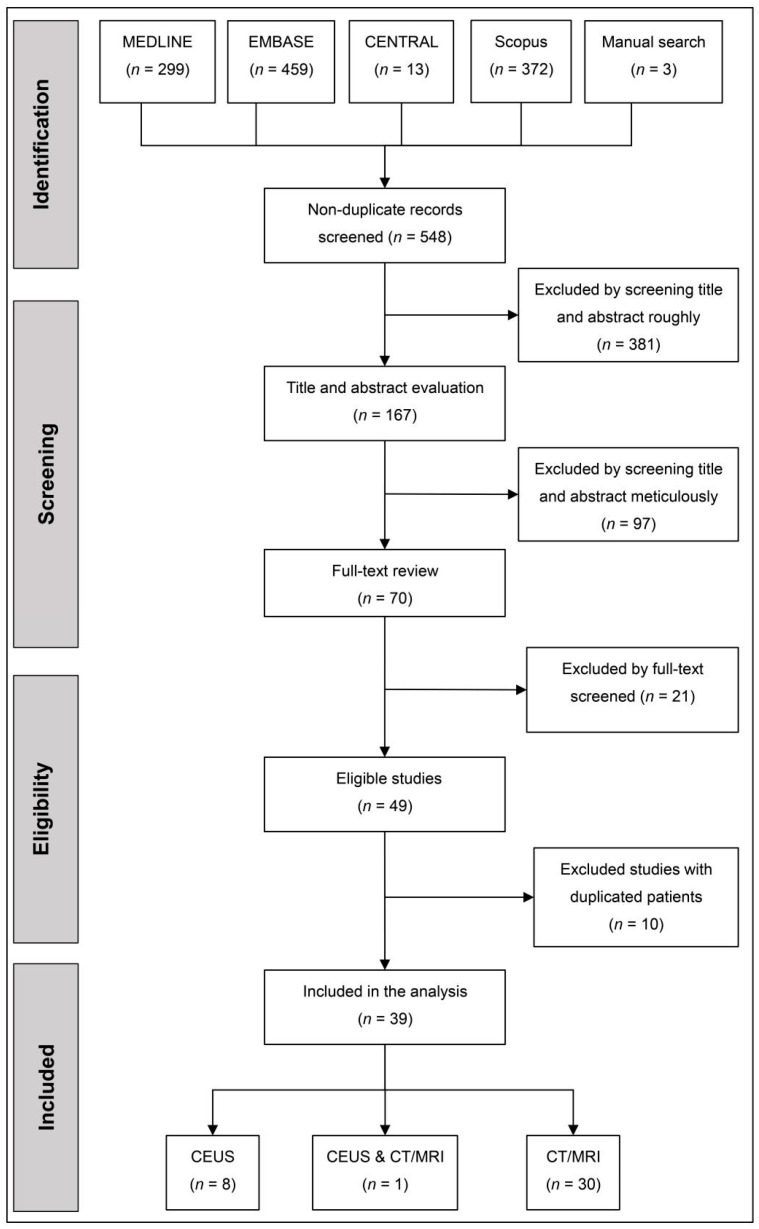
The flow diagram of study selection. CENTRAL, cochrane central register of controlled trials. CEUS, contrast-enhanced ultrasound.

Five hundred and forty-eight studies were screened, of which 39 records were eventually included. The flow diagram of the study selection is shown in Figure 1 and the details of excluded studies are given in Supplementary Table S3. Among 39 studies, nine records [20,21,22,23,24,25,26,27,28] demonstrated the accuracy for HCC diagnosis of CEUS LR5, and 31 [20,29,30,31,32,33,34,35,36,37,38,39,40,41,42,43,44,45,46,47,48,49,50,51,52,53,54,55,56,57,58] evaluated that of CT/MRI LR5, while eight records [21,22,23,24,25,26,27,28] evaluated the performance of CEUS LRM for characterizing OM and 19 reported [29,30,31,32,33,34,35,36,37,38,39,40,41,42,43,44,45,46,47] that of CT/MRI LRM.

### 3.2. Study Characteristics

The baseline characteristics of the included studies are shown in Table 1. Eventually, we included 5179 lesions (3841 HCCs, 456 OMs, and 884 benign lesions) for CEUS and 7908 lesions (5443 HCC, 762 OM, and 1713 benign lesions) for CT/MRI (Table 2).

### 3.3. Quality Assessment 

The overall risk of bias of the 39 studies was low to moderate, shown in Supplementary Figure S1. Patient selection was the most significant source of bias. Thirteen studies were identified as being at high risk of bias in the domain of patient selection because they did not avoid a case-control design and/ or enroll lesions diagnosed by composite clinical reference standards. Studies that employed LR5 of another imaging modality without follow-up as a reference standard were considered at high risk of bias in the domain of reference standard.

### 3.4. Diagnostic Performance of LR5 for Diagnosing HCC

**Figure 2:**
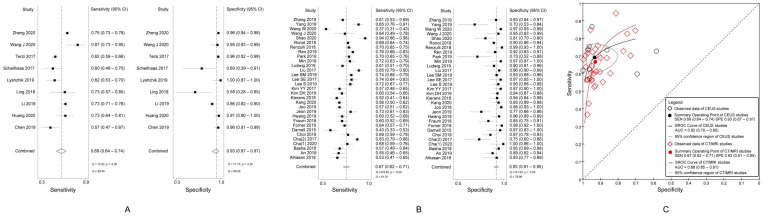
(**A**) Sensitivity and specificity of CEUS LR5 for diagnosing HCC; (**B**) Sensitivity and specificity of CT/MRI LR5 for diagnosing HCC; (**C**) Summary receiver operating characteristic curves of CEUS LR5 and CT/MRI LR5 for diagnosing HCC. CEUS, contrast-enhanced ultrasound; LR5, Liver Imaging Reporting and Data System category 5; HCC, hepatocellular carcinoma.

The pooled sensitivity and specificity of CEUS LR5 for diagnosing HCC were, respectively, 69% (95% CI, 64%–74%; *I*^2^ = 89.45%; *p* < 0.01) and 93% (95% CI, 87%–97%; *I*^2^ = 88.85%; *p* < 0.01) (Figure 2A).

The pooled sensitivity was 67% (95% CI, 62–71%; *I*^2^ = 91.01%; *p* < 0.01) and the specificity, 93% (95% CI, 91–95%; *I*^2^ = 78.86%; *p* < 0.01) of CT/MRI LR5 for diagnosing HCC (Figure 2B).

There was no significant difference in the DOR of the LR5 for HCC diagnosis between CEUS (31.83, 95% CI, 14.04–72.15) and CT/MRI (26.55, 95% CI, 19.81–35.56) (*p* = 0.55). The area under the summary receiver operating characteristic (SROC) curve of CEUS LR5 for HCC and that of CT/MRI LR5 were 0.82 (95% CI, 0.79–0.85) and 0.88 (95% CI, 0.85–0.91), respectively (Figure 2C).

### 3.5. Diagnostic Performance of LRM for Characterizing OM

**Figure 3:**
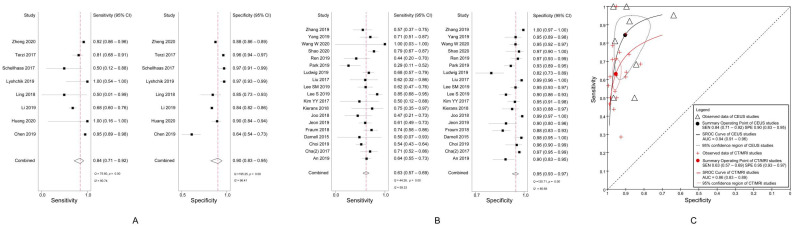
(**A**) Sensitivity and specificity of CEUS LRM for characterizing OM; (**B**) Sensitivity and specificity of CT/MRI LRM for characterizing OM; (**C**) Summary receiver operating characteristic curves of CEUS LRM and CT/MRI LRM for characterizing OM. CEUS, contrast-enhanced ultrasound; LRM, Liver Imaging Reporting and Data System category M; OM, other non-HCC malignancies.

A pooled analysis showed a sensitivity of 84% (95% CI, 71–92%; *I*^2^ = 90.74%; *p* < 0.01) and a specificity of 90% (95% CI, 83–95%; *I*^2^ = 96.41%; *p* < 0.01) for characterizing OM in respect of CEUS LRM (Figure 3A).

The pooled sensitivity was 63% (95% CI, 57–69%; *I*^2^ = 59.33%; *p* < 0.01) and the specificity, 95% (95% CI, 93–97%; *I*^2^ = 85.68%; *p* < 0.01) of CT/MRI LRM for characterizing OM (Figure 3B).

The DOR of CEUS LRM for characterizing OM was 50.59 (95% CI, 22.16–115.52), and that of CT/MRI LRM was 36.06 (95% CI, 23.70–54.87) (*p* = 0.34). Analysis of the area under the SROC curves showed that the accuracy of CEUS LRM for characterizing OM and that of CT/MRI was 0.94 (95% CI, 0.91–0.96) and 0.86 (95% CI, 0.83–0.89), respectively (Figure 3C).

### 3.6. Meta-Regression Analysis

Meta-regression analyses of the sensitivity and specificity of CEUS LR5 for diagnosing HCC and CEUS LRM for characterizing OM are demonstrated in Supplementary Table S4. Among six covariates, country, average lesion size and proportion of OM were the factors that contributed to the heterogeneity of the sensitivity of CEUS LR5 for diagnosing HCC. Regarding the reference standard, the specificity of CEUS LR5 for diagnosing HCC was higher for studies using a mixed reference standard as a reference standard than for those that employed only pathology (96% vs. 89%; *p* = 0.06). Country, study design, proportion of OM, and reference standard were the factors that significantly influenced the heterogeneity of the specificity of the CEUS LRM category for characterizing OM.

Meta-regression analyses of the sensitivity and specificity of the CT/MRI LR5 category for diagnosing HCC and the CT/MRI LRM category for characterizing OM were also performed (Supplementary Table S5). We noted that the pooled sensitivity was 67% (95% CI, 62–72%) and the specificity, 93% (95% CI, 91–96%) of LR5 for diagnosing HCC with CT/MRI LI-RAD v2017/2018, superior to those with v2014 (sensitivity, 65%, 95% CI, 56–74%; specificity, 92%, 95% CI, 88–96%; *p* = 0.05). As for average size, the sensitivity of CT/MRI LI-RADS for diagnosing HCC and characterizing OM was reduced when small lesions were enrolled (Sensitivity of LR5 for diagnosing HCC, 61% vs. 68%; Sensitivity of LRM for characterizing OM, 57% vs. 64%), similar to the findings observed in CEUS studies. Besides, the index test was one of the factors that significantly influenced the heterogeneity of the sensitivity and specificity for both HCC and non-HCC malignancies with CT/MRI LI-RADS.

### 3.7. Publication Bias

No obvious publication bias was identified through the funnel plot and Egger’s test (*p* > 0.05), shown in Supplementary Figure S2.

## 4. Discussion

The meta-analysis included more than 5000 lesions in each LI-RADS. A pooled analysis revealed a sensitivity of 69% and a specificity of 93% of CEUS LR5 and a sensitivity of 67% and a specificity of 93% of CT/MRI LR5 for diagnosing HCC. This study also indicated that the pooled sensitivity and specificity for characterizing OM with respect to CEUS LRM were 84% and 90%, respectively, and those of CT/MRI LRM were 63% and 95%. 

Our meta-analysis found that the pooled sensitivity and specificity for diagnosing HCC of CEUS LR5 was 69% and 93%, respectively, which was quite similar to those summary estimates of CT/MRI LR5 (sensitivity 67% and specificity 93%). A study conducted by Shin et al. [16] suggested that the diagnostic performances for diagnosing HCC of LI-RADS with CEUS and CT/MRI can be compared. In our study, there was no statistical difference between CEUS LR5 and CT/MRI LR5 in the overall diagnostic accuracy for diagnosing HCC (*p* = 0.55), but a specificity of 93% for HCC diagnosis of LR5 was lower than that preconceived in the LI-RADS algorithm (definitely HCC). 

Patients with ICC and HCC-CCA have a poorer prognosis and fewer treatment options than those patients with HCC. Therefore, distinguishing OM from HCC will be of great significance for making clinical decisions. In our meta-analysis, CEUS LRM showed high sensitivity (84%) and high specificity (90%) for characterizing OM, while moderate sensitivity (63%) and high specificity (95%) were observed in CT/MRI LRM. The overall diagnostic accuracy of CEUS LRM was higher than that of CT/MRI LRM without significant difference (*p* = 0.34). In other words, compared to CT/MRI, CEUS did not increase the risk of misdiagnosing OM as HCC. This finding is not in agreement with the results of some previous studies which indicated CEUS was associated with a high risk of misdiagnosis [7].

CEUS is different from CT/MRI in the following aspects [59]: (a) physicochemical properties of contrast agent used, (b) method to obtain images (for CEUS, dynamic real-time imaging; for CT/MRI, static imaging) and (c) criteria adopted to assign the LI-RADS category to a liver nodule. Based on the above, CEUS may provide additional information of focal liver nodules to CT/MRI. We suggested that CEUS is useful in assisting to provide an accurate diagnosis for patients at risk of HCC and a multimodality approach might be needed to improve the diagnostic performance of characterizing HCC and OM. 

Regarding the baseline characteristics of the included studies, we noted that most were from Asian countries, followed by European and North American countries. Although the epidemiological features and imaging characteristics of HCC in non-Asian countries were different from that in Asian countries, CEUS LI-RADS and CT/MRI LI-RADS introduced by the American association were also suitable to be applied in Asian countries where HBV is endemic. Patients with HBV infection are at risk for HCC even without cirrhosis. In a cirrhotic liver, we need to differentiate HCC from regenerating nodules, and for patients with HBV infection in the absence of cirrhosis, we do not need to do so. This might lead to superior diagnostic performance for HCC. However, the prevalence of cirrhosis in the whole sample was lower than that in western countries, so the generalizability of these results should be further assessed. There are only a limited number of studies evaluating the diagnostic performance of CEUS LI-RADS.The main reasons are as follows: (a) The CEUS LI-RADS was officially issued in 2017, while CT/MRI LI-RADS was first released in 2011 and multiple revisions were updated after that. In other words, CEUS LI-RADS has a short time of application worldwide in comparison to CT/MRI LI-RADS. (b) Some centers adopted other CEUS-based standardized algorithms (such as ESCULAP, EFSUMB) in clinical practice instead of LI-RADS. Among 39 included records, only eight were prospective studies, eight were multicenter studies and twenty-nine employed a cohort study design. More prospective, multicenter cohort researches that focus on the LI-RADS algorithm are expected to be published in the future.

QUADAS-2 was used to assess the methodological quality of the included studies. More than half of the studies had at least one domain at high risk of bias. In our study, case-control studies were viewed to be inferior to those cohort studies since the percentage of HCC and OM depended on the researchers. Using pathology as a reference standard exclusively might introduce verification bias because lesions receiving pathological assessment are more inclined to be malignant and be assigned to higher LI-RADS categories. There is likely to be incorporation bias for studies employing LR5 of another imaging modality without follow-up as a reference standard [14].

To a certain extent, there were clinical and methodological similarities between studies because of cautious study selection and methodological quality assessment, but there was still statistical heterogeneity between the included studies. The meta-regression analysis showed that the specificity of the CEUS LR5 for diagnosing HCC in the studies employing merely pathology as a reference standard was significantly lower than those employing a mixed reference standard (89% vs. 96%, *p =* 0.03). A similar finding was observed in the CT/MRI studies. HCC with typical imaging features can be easily diagnosed by composite imaging, whereas atypical HCC was difficult to be definitely diagnosed by imaging and should be biopsied. Studies using only a pathological reference standard might contain more atypical HCC than those using a mixed reference standard, which may explain their lower specificity for characterizing HCC. We also noted that when small lesions were enrolled, the sensitivity for diagnosing HCC and characterizing OM was reduced both at CEUS and CT/MRI. Small liver nodules usually possess atypical imaging features, so that it remains a challenge to distinguish small HCC and OM from other focal liver lesions, especially under the background of cirrhosis. 

Comparing CT/MRI LI-RADS v2014, v2017 and v2018 made major revisions to applicable patients, diagnostic categories, threshold growth definition, LRM criteria, and ancillary features. The pooled sensitivity was 67% and the specificity was 93% of LR5 for diagnosing HCC with CT/MRI LI-RAD. v2017/2018 was superior to those with v2014 (sensitivity, 65%; specificity, 92%; *p* = 0.05). It seemed that the revisions significantly improved the diagnostic performance for diagnosing HCC. The index test significantly influenced the heterogeneity of the sensitivity and specificity for both HCC and non-HCC malignancies with CT/MRI LI-RADS. So we performed three meta-analyses that compared the different techniques (CEUS, CT, MRI) two by two after removing four studies [30,33,40,41] which didn’t evaluate the diagnostic accuracy at CT and MRI imaging separately (Supplementary Table S6).

We tried to answer the question of whether CEUS is qualified to characterize HCC and OM accurately, which were closely relevant to the management of liver nodules. There were some limitations. First, apparent heterogeneity was identified in our meta-analysis. To seek the covariates that contributed to the heterogeneity, a meta-regression analysis was performed, but the heterogeneity still cannot be ignored. Second, an adjusted indirect comparison was used instead of direct comparison, mainly due to insufficient evidence comparing the diagnostic accuracy of CEUS LI-RADS and CT/MRI LI-RADS head-to-head. A study reported that adjusted indirect comparison might be less biased than direct comparison [60]. Finally, we did not perform subgroup analyses based on major and ancillary features of the LI-RADS because of insufficient data. Thus, our results should be confirmed by more evidence. Considering the limitations, more high-quality direct comparisons between CEUS LI-RADS and CT/MRI LI-RADS are required to update this meta-analysis. At present, we have been working on a multicenter study comparing the diagnostic accuracy of CEUS LI-RADS and CT/MRI LI-RADS for characterizing HCC and OM directly with a large sample size. We also keep a concurring opinion with the study [14] that an individual participant meta-analysis is needed. 

In conclusion, both CEUS LR5 and CT/MRI LR5 showed moderate sensitivity and high specificity for diagnosing HCC. CEUS LRM showed high sensitivity and high specificity for characterizing OM, while CT/MRI LRM showed moderate sensitivity and high specificity.

## Figures and Tables

**Table 1:** Main characteristics of the included studies.

Reference	Background				Patients			Index Test	
	Country	Centre	Study Type	Study Design	No. of Patients	Average Age (y)	Male, %	Imaging Modality	LI-RADS Version
Chen et al., 2019 [21]	China	Single	Retrospective	Case-control	210	54.5	77.6	CEUS	2017
Huang et al., 2020 [22]	China	Single	Retrospective	Cohort	172	51.8	79.1	CEUS	2017
Li et al., 2019 [23]	China	Single	Retrospective	Cohort	1366	52.3	80.3	CEUS	2017
Ling et al., 2018 [24]	China	Single	Retrospective	NR	56	52.5	82.1	CEUS	2017
Lyshchik et al., 2019 [28]	USA	Multiple	Prospective	Cohort	NR	NR	NR	CEUS	2017
Schellhaas et al., 2017 [25]	Germany	Single	Prospective	Cohort	100	66.1	85.0	CEUS	2016
Terzi et al., 2017 [26]	Italy	Multiple	Retrospective	Cohort	848	70	53.9	CEUS	2016
Zheng et al., 2020 [27]	China	Single	Retrospective	Cohort	1826	54	89.9	CEUS	2017
Wang et al., 2020 [20]	China	Single	Retrospective	Cohort	63	56	74.6	CEUS	2017
								ECA-MRI	2018
Alhasan et al., 2018 [48]	Canada	Single	Retrospective	Cohort	59	63.2	76.3	CT	2017
An et al., 2019 [29]	Korea	Multiple	Retrospective	Case-control	217	59	76.5	CT, HBA-MRI	2014
Basha et al., 2018 [49]	Egypt	Multiple	Prospective	Cohort	240	61.5	55.8	CT, ECA-MRI	2014
Cha(1) et al., 2020 [50]	Korea	Single	Prospective	Cohort	122	55	82.8	ECA-MRI, HBA-MRI	2018
Cha(2) et al., 2017 [30]	Korea	Single	Retrospective	Cohort	421	57	72.0	CT, HBA-MRI ^a^	2014
Choi et al., 2019 [31]	Korea	Single	Retrospective	Case-control	194	57	79.9	HBA-MRI	2017
Darnell et al., 2015 [32]	Spain	Single	Retrospective	Cohort	133	64	50.9	ECA-MRI	2014
Forner et al., 2019 [51]	Spain	Single	Retrospective	NR	262	NR	NR	MRI	2018
Fraum et al., 2018 [33]	USA	Single	Prospective	Cohort	220	60.3	NR	CT, ECA-MRI, HBA-MRI ^a^	2014
Hwang et al., 2019 [52]	Korea	Single	Retrospective	Cohort	177	58	89.3	HBA-MRI	2018
Jeon et al., 2019 [34]	Korea	Single	Retrospective	Case-control	140	56	71.7	HBA-MRI	2017
Joo et al., 2018 [35]	Korea	Single	Retrospective	NR	288	NR	NR	HBA-MRI	2017
Kang et al., 2020 [53]	Korea	Single	Retrospective	Cohort	266	61.3	81.2	HBA-MRI	2018
Kierans et al., 2018 [36]	USA	Multiple	Retrospective	Cohort	114	57	65.8	ECA-MRI, HBA-MRI	2017
Kim DH et al., 2019 [54]	Korea	Single	Prospective	Cohort	258	61	81.4	HBA-MRI	2018
Kim YY et al., 2017 [37]	Korea	Single	Retrospective	Cohort	143	58	83.9	HBA-MRI	2014
Lee S et al., 2019 [39]	Korea	Single	Retrospective	Cohort	298	57.4	72.1	ECA-MRI, HBA-MRI	2018
Lee SE et al., 2017 [55]	Korea	Single	Retrospective	Cohort	103	61	74.8	ECA-MRI	2017
Lee SM 2019 [38]	Korea	Single	Retrospective	Cohort	387	59	78.8	HBA-MRI	2017, 2018
Liu et al., 2017 [40]	China	Single	Retrospective	NR	249	51	85.5	CT, MRI ^a^	2014
Ludwig et al., 2019 [41]	USA	Multiple	Retrospective	Cohort	178	61.9	77.5	CT, ECA-MRI, HBA-MRI ^a^	2018
Min et al., 2019 [56]	Korea	Single	Prospective	Cohort	125	55.3	81.6	CT, ECA-MRI, HBA-MRI	2018
Park et al., 2019 [42]	Korea	Multiple	Retrospective	Cohort	267	56.2	77.9	HBA-MRI	2018
Ren et al., 2019 [43]	China	Single	Retrospective	Cohort	181	56.4	77.9	ECA-MRI	2017, 2018
Renzulli et al., 2018 [57]	Italy	Single	Retrospective	Cohort	228	63.7	79.4	HBA-MRI	2017
Ronot et al., 2018 [58]	France	Multiple	Prospective	Cohort	442	61.9	77.6	CT, ECA-MRI	2014
Shao et al., 2020 [44]	China	Single	Retrospective	Case-control	140	48	85.7	ECA-MRI, HBA-MRI	2018
Wang W et al., 2020 [45]	China	Single	Retrospective	Cohort	204	55	89.2	HBA-MRI	2018
Yang et al., 2019 [46]	China	Single	Retrospective	NR	130	51.5	76.2	HBA-MRI	2018
Zhang et al., 2019 [47]	China	Single	Retrospective	Cohort	203	50.3	77.3	HBA-MRI	2017

Abbreviations: LI-RADS, Liver Imaging Reporting and Data System. CEUS, contrast-enhanced ultrasound. CT, computed tomography. MRI, magnetic resonance imaging. ECA, extracellular contrast agents. HBA, hepatobiliary agents. NR, not reported. ^a^, these studies did not evaluate the diagnostic accuracy at CT and MRI separately.

**Table 2:** Lesions and their reference standards.

Reference	Patients		Lesions and Reference Standards							
	*n*	Cirrhosis, %	*n*	Pathology, %	Average Size (Range, mm)	HCC, *n*	OM, *n*	Benign Lesion, *n*	Interval from Index Test to Pathology	Interval from Index Test to Follow-Up
Chen et al., 2019 [21]	210	NR ^£^	210	100.0	NR	105 ^†^	105 ^†^	0	NR	N/A
Huang et al., 2020 [22]	172	NR	175	70.9	16.1 (8–20)	105 ^†^^,‡,§^	2 ^†^	68 ^†^^,‡,§^	(13 ± 7) d	NR
Li et al., 2019 [23]	1366	37.5	1366	100.0	47 (5–200)	985 ^†^	139 ^†^	242 ^†^	NR	N/A
Ling et al., 2018 [24]	56	8.9	56	100.0	17.1 (14.3–20)	44 ^†^	2 ^†^	10 ^†^	NR	N/A
Lyshchik et al., 2019 [28]	NR	NR	162	NR	24 (NR)	136 ^†^^,‡,§^	6 ^NR^	20 ^NR^	NR	NR
Schellhaas et al., 2017 [25]	100	4.0	100	63.0	52.2 (10–290)	87 ^†^^,‡^	6 ^†^	7 ^†^^,§^	≤3 m	≥6 m
Terzi et al., 2017 [26]	848	100.0	1066	46.9	20 (5–150)	820 ^†^^,‡^	53 ^†^	133 ^†^^,‡^	NR	N/A
Wang J et al., 2020 [20]	63	96.8	84	100.0	35 (9–183)	45 ^†^	4 ^†^	35 ^†^	NR	N/A
Zheng et al., 2020 [27]	1826	86.3	2020	35.5	NR	1514 ^†^^,‡,§^	138 ^†^^,‡^	368 ^†^^,‡,§^	NR	≥12 m
Alhasan et al., 2018 [48]	59	93.2	104	41.3	30.1 (6–110)	72 ^†^^,‡,§^	4 ^†^	38 ^†^^,‡,§^	HCC:(137 ± 206) d Benign lesions:(251 ± 441) d	≥6 m
An et al., 2019 [29]	217	NR	231	100.0	21 (3−133)	114 ^†^	58 ^†^	59 ^†^	≤2 m	N/A
Basha et al., 2018 [49]	240	NR	296	63.2	NR (≤50)	192 ^†^^,§^	9 ^†^	95 ^†^^,§^	NR	NR
Cha(1) et al., 2020 [50]	122	59.8	147	95.9	21 (6–50)	122 ^†^	10 ^†^	15 ^†^^,§^	≤1 m	NR
Cha(2) et al., 2017 [30]	421	NR	445	100.0	25 (8–49)	397 ^†^	31 ^†^	17 ^†^	≤2 m	N/A
Choi et al., 2019 [31]	194	100.0	194	100.0	32 (NR)	97 ^†^	97 ^†^	0	≤3 m	N/A
Darnell et al., 2015 [32]	133	100.0	133	NR	NR (5–20)	102 ^†^	4 ^†^	27 ^†^^,‡,§^	NR	NR
Forner et al., 2019 [51]	262	100.0	262	NR	NR (≤20)	197 ^†^^,‡,§^	7 ^†^	58 ^†^^,‡,§^	NR	NR
Fraum et al., 2018 [33]	220	66.4	220	100.0	NR	136 ^†^	42 ^†^	42 ^†^	NR	N/A
Hwang et al., 2019 [52]	177	NR	241	71.0	19.7 (2.3–48.2)	149 ^†^	6 ^†^	86 ^†^^,§^	≤6 m	≥24 m
Jeon et al., 2019 [34]	140	12.1	140	100.0	39.4 (NR)	70 ^†^	70 ^†^	0	≤2 m	N/A
Joo et al., 2018 [35]	288	57.6 ^Ψ^	387	39.3	NR	292 ^†^^,‡^	15 ^†^	80 ^†^^,‡,§^	≤2 m	≥6 m
Kang et al., 2020 [53]	266	NR	385	62.9	NR (5–30)	283 ^†^^,§^	18 ^†^	84 ^†^^,§^	NR	≥24 m
Kierans et al., 2018 [36]	114	NR ^₴^	144	71.5	NR	82 ^†^	8 ^†^	54 ^†^^,§^	≤4 m	≥24 m
Kim DH et al., 2019 [54]	258	NR	372	63.4	NR (10–30)	273 ^†^^,§^	18 ^†^	81 ^†^^,§^	NR	≥24 m
Kim YY et al., 2017 [37]	143	NR	202	61.9	NR	129 ^†^^,§^	6 ^†^	67 ^†^^,§^	35 (1–461) d	≥18 m
Lee S et al., 2019 [39]	298	63.1	382	85.6	24 (15–34)	286 ^†^	33 ^†^	63 ^†^^,‡,§^	≤3 m	≥24 m
Lee SE et al., 2017 [55]	103	NR	133	75.2	22.2 (4.9–192)	107 ^†^^,§^	3 ^†^	23 ^†^^,§^	NR	≥18 m
Lee SM et al., 2019 [38]	387	74.2 ^Ψ^	422	70.4	24 (NR)	234 ^†^	45 ^†^	143 ^†^^,§^	≤2 m	≥24 m
Liu et al., 2017 [40]	249	NR	297	64.3	22 (3–146)	178 ^†^	13 ^†^	106 ^†^^,§^	≤1 m	≥24 m
Ludwig et al., 2019 [41]	178	97.8 ^Ψ^	178	100.0	35 (14–190)	105 ^†^	73 ^†^	0	HCC:189d; OM:89d	N/A
Min et al., 2019 [56]	125	52.8 ^£^	163	89.6	20.7 (9–40)	124 ^†^	13 ^†^	26 ^†^^,‡,§^	≤1 m	NR
Park et al., 2019 [42]	267	100.0	306	100.0	19 (5–30)	280 ^†^	21 ^†^	5 ^†^	NR	N/A
Ren et al., 2019 [43]	181	32.6	217	59.0	30.5 (NR)	146 ^†^^,§^	16 ^†^	55 ^†^^,‡,§^	NR	≥24 m
Renzulli et al., 2018 [57]	228	NR	420	38.3	16.7 (11–150)	342 ^†^^,‡,§^	8 ^†^	69 ^†^^,§^	NR	≥24 m
Ronot et al., 2018 [58]	442	100.0 ^Ψ^	595	NR	18 (10–30)	341 ^†,‡,§^	8 ^¶^	246 ^¶^	≤6 m	≥6 m
Shao et al., 2020 [44]	140	NR	140	100.0	48 (10–120)	70 ^†^	70 ^†^	0	≤6 m	N/A
Wang W et al., 2020 [45]	204	100.0	373	NR	14 (5.5–19)	256 ^†^^,‡^	1 ^†^	116 ^†^^,§^	NR	≥24 m
Yang et al., 2019 [46]	130	NR	134	100.0	49.2 (NR)	97 ^†^	29 ^†^	8 ^†^	≤1 m	N/A
Zhang et al., 2019 [47]	203	NR	245	81.6	53 (11–128)	165 ^†^	30 ^†^	50 ^†^^,§^	NR	≥24 m

Abbreviations: HCC, hepatocellular carcinoma. OM, other non-HCC malignancies. ^£^, cirrhosis of any origin confirmed by pathological examination. ^Ψ^, cirrhosis confirmed by pathological assessment or clinical diagnostic criteria. ^₴^, clinically diagnosed cirrhosis. ^†^, pathological analysis. ^‡^, contrast-enhanced CT or MRI. ^§^, follow-up. ^¶^, did not meet the criteria for HCC. d, days. m, months. n, number. NR, not reported. N/A, not applicable.

## Data Availability

All analyses were based on previously published studies. No new data were created in this study. Data sharing is not applicable to this article.

## Supplementary Materials

The following are available online at https://www.mdpi.com/2075-4418/11/2/323/s1, Figure S1. Methodological quality assessment using the Quality Assessment of Diagnostic Accuracy Studies 2 tool; Figure S2. Deek’s funnel plot and Egger’s test to evaluate the publication bias of (A) studies evaluating the diagnostic performance of CEUS LR5 for HCC diagnosis, (B) studies evaluating the diagnostic performance of CT/MRI LR5 for HCC diagnosis, (C) studies evaluating the diagnostic performance of CEUS LRM for characterizing OM, and (D) studies evaluating the diagnostic performance of CT/MRI LRM for characterizing OM. CEUS, contrast-enhanced ultrasound; HCC, hepatocellular carcinoma; OM, other non-HCC malignancies; LR5, Liver Imaging Reporting and Data System category 5; LRM, Liver Imaging Reporting and Data System category M; ESS, effective sample sizes; *p* > 0.05, no obvious publication bias was identified; Table S1. Details of search strategy; Table S2. Data form; Table S3. Details of excluded studies; Table S4. The results of meta-regression of the sensitivity and specificity of the CEUS LR5 for diagnosing HCC and the CEUS LRM for characterizing OM; Table S5. The results of meta-regression of the sensitivity and specificity of the CT/MRI LR5 for diagnosing HCC and the CT/MRI LRM for characterizing OM; Table S6. The diagnostic performance of LR5 for diagnosing HCC and LRM for characterizing OM with CEUS, CT and MRI separately.

## Abbreviations

AASLD	American Association for the Study of Liver Disease
ACR	American College of Radiology
CEUS	Contrast-enhanced ultrasound
DOR	Diagnostic odds ratio
HCC	Hepatocellular carcinoma
HCC-CCA	Mixed hepatocellular and cholangiocarcinoma
ICC	Intrahepatic cholangiocarcinoma
LI-RADS	Liver Imaging Reporting and Data System
LR5	LI-RADS category 5
LRM	LI-RADS category M
OM	Other non-HCC malignancies
QUADAS-2	Quality Assessment of Diagnostic Accuracy Studies 2
SROC	Summary receiver operating characteristic
