# Supplementary material for: Clinical Application of Liver Imaging Reporting and Data System for Characterizing Liver Neoplasms: A Meta-Analysis
Source: Diagnostics (Basel). 2021 Feb 17;11(2):323. doi: 10.3390/diagnostics11020323 (PMC7921912; doi:10.3390/diagnostics11020323)
Supplement: Supplementary file 1 [file diagnostics-11-00323-s001.zip › supplementary file/Supplementary Table.docx]

**Supplementary Table 1 Details of search strategy**

| **Database：EMBASE (**<https://www.embase.com/>) | | |
| --- | --- | --- |
| History | Search query | Entry |
| #1 | 'hepatocellular carcinoma cell line'/exp OR 'bile duct carcinoma'/exp OR 'liver tumor'/exp | 330,455 |
| #2 | (hepatocellular AND carcinoma*) OR (hepatocellular AND neoplasm*) OR (hepatocellular AND cancer*) OR HCC OR cholangiocarcinoma* OR ICC OR (liver AND lesion*) OR (liver AND nodule*) [All Fields] | 278,954 |
| #3 | LR-1 OR LR-2 OR LR-3 OR LR-4 OR LR-5 OR LR-5V OR LR-M OR LM-OM OR LR-TIV OR LR1 OR LR2 OR LR3 OR LR4 OR LR5 OR LRM OR LROM OR LR5V OR LRTIV [All Fields] | 3,693 |
| #4 | #1 OR #2 | 446,240 |
| #5 | #3 AND #4 | 243 |
| #6 | 'LI-RADS' OR ‘LIRADS’ OR 'liver imaging reporting and data system' | 408 |
| #7 | #5 OR #6 AND [2014-2020]/py | 459 |
| **Database：Ovid CENTRAL (**<http://ovidsp.ovid.com/>) | | |
| History | Search query | Entry |
| #1 | Carcinoma, Hepatocellular/ or Cholangiocarcinoma/ | 1991 |
| #2 | (hepatocellular carcinoma* or hepatocellular neoplasm* or hepatocellular cancer* or HCC or cholangiocarcinoma* or ICC or liver nodule* or liver lesion*). kf,kw,tw. | 6909 |
| #3 | Liver imaging. kf,kw,tw. | 63 |
| #4 | (LR-1 or LR-2 or LR-3 or LR-4 or LR-5 or LR-5V or LR-M or LM-OM or LR-TIV or LR1 or LR2 or LR3 or LR4 or LR5 or LRM or LROM or LR5V or LRTIV). tw. | 466 |
| #5 | (LI-RADS or LIRADS). kf,kw,tw. | 9 |
| #6 | (liver imaging reporting and data system). kw,tw. | 5 |
| #7 | #1 or #2 or #3 | 7227 |
| #8 | #4 and #7 | 7 |
| #9 | #5 or #6 | 9 |
| #10 | #8 or #9 and limit 2 to yr="2014 -Current" | 13 |
| **Database： Ovid MEDLINE (**<http://ovidsp.ovid.com/>) | | |
| History | Search query | Entry |
| #1 | Carcinoma, Hepatocellular/ or Cholangiocarcinoma/ | 89870 |
| #2 | (hepatocellular carcinoma* or hepatocellular neoplasm* or hepatocellular cancer* or HCC or cholangiocarcinoma* or ICC or liver nodule* or liver lesion*). kf,kw,tw. | 109269 |
| #3 | Liver imaging. kf,kw,tw. | 1032 |
| #4 | (LR-1 or LR-2 or LR-3 or LR-4 or LR-5 or LR-5V or LR-M or LM-OM or LR-TIV or LR1 or LR2 or LR3 or LR4 or LR5 or LRM or LROM or LR5V or LRTIV). tw. | 1502 |
| #5 | (LI-RADS or LIRADS). kf,kw,tw. | 273 |
| #6 | (liver imaging reporting and data system). kw,tw. | 208 |
| #7 | #1 or #2 or #3 | 135576 |
| #8 | #4 and #7 | 122 |
| #9 | #5 or #6 | 295 |
| #10 | #8 or #9 and limit 2 to yr="2014 -Current" | 299 |
| **Database： Scopus (**<https://www.scopus.com>) | | |
| Search query | | Entry |
| ( ( ( ALL ( "hepatocellular carcinoma*" OR " hepatocellular neoplasm*" OR " hepatocellular cancer*" OR "HCC" OR "ICC" OR "cholangiocarcinoma" OR "liver lesion*" OR "liver nodule*" ) ) OR ( TITLE-ABS-KEY ( "liver imaging" ) ) ) AND ( ALL (LI-1 OR LI-2 OR LI-3 OR LI-4 OR LI-5 OR LI-5V OR LI-M OR LI-OM OR LI-TIV OR LI1 OR LI2 OR LI3 OR LI4 OR LI5 OR LIM OR LIOM OR LI5V OR LITIV ) ) ) OR TITLE-ABS-KEY ( "LIRADS" OR "LI-RADS" OR "liver imaging reporting and data system" ) AND PUBYEAR AFT 2014 | | 372 |

**Supplementary Table 2 Data form**

| **Background** | |
| --- | --- |
| Name of Primary study |  |
| First author |  |
| Country |  |
| Affiliation |  |
| Journal |  |
| Year |  |
| Funding |  |
| Conflicts of interest |  |
| **Study design** | |
| Study period |  |
| Study design (retrospective or prospective) |  |
| Study type (case-control or cohort) |  |
| Subject enrollment (consecutive or selective) |  |
| Image reviewer (multiple or single; independently or consensus; and working experience) |  |
| Blind to other imaging or examinations or pathology？(YES/NO/UNCLEAR) |  |
| **Patients** | |
| Number of patients |  |
| Male(n/%) |  |
| Average age |  |
| Cirrhosis(n/%) |  |
| **Lesions** | |
| Number of lesions | |
| Number of HCC |  |
| Number of other non-HCC malignancies (OM) |  |
| Number of benign |  |
| Lesion limited? (YES/NO） |  |
| Average lesion size (mm) |  |
| **Reference standards** | |
| Interval between index test and pathological assessment |  |
| Interval between index test and follow-up |  |
| Reference standard for HCC |  |
| Reference standard for OM |  |
| Reference standard for benign |  |
| Number of patients with pathological assessment (%) |  |
| **Index tests** | |
| Index text (CEUS/ CT/MRI) |  |
| LI-RADS version |  |
| 1. **CEUS** | |
| Contrast agent |  |
| Quantity of contrast agent |  |
| Device/Probe |  |
| Mechanical index |  |
| 1. **CT** | |
| Contrast agent |  |
| Quantity of contrast agent |  |
| Rate(ml/s) |  |
| Device |  |
| Detector (16/64/128) |  |
| Phases |  |
| MRI | |
| Contrast agent |  |
| Quantity of contrast agent |  |
| Rate(ml/s) |  |
| Devices |  |
| MR intensity (3.0T/1.5T) |  |
| DWI (YES/NO/UNCLEAR) |  |
| Parameters (CLEAR/UNCLEAR) |  |
| LR1 | |
| Number of lesions |  |
| HCC(n/%) |  |
| OM(n/%) |  |
| Benign (n/%) |  |
| LR2 | |
| Number of lesions |  |
| HCC(n/%) |  |
| OM(n/%) |  |
| Benign (n/%) |  |
| LR3 | |
| Number of lesions |  |
| HCC(n/%) |  |
| OM(n/%) |  |
| Benign (n/%) |  |
| LR3 | |
| Number of lesions |  |
| HCC(n/%) |  |
| OM(n/%) |  |
| Benign (n/%) |  |
| LR4 | |
| Number of lesions |  |
| HCC(n/%) |  |
| OM(n/%) |  |
| Benign (n/%) |  |
| LR5 | |
| Number of lesions |  |
| HCC(n/%) |  |
| OM(n/%) |  |
| Benign (n/%) |  |
| LRM | |
| Number of lesions |  |
| HCC(n/%) |  |
| OM(n/%) |  |
| Benign (n/%) |  |
| The LR5 category for HCC diagnosis |  |
| Sensitivity |  |
| Specificity |  |
| PPV |  |
| NPV |  |
| True positive (TP) |  |
| False positive (FP) |  |
| True negative (TN) |  |
| False negative (FN) |  |
| The LRM category for charactering OM |  |
| Sensitivity |  |
| Specificity |  |
| PPV |  |
| NPV |  |
| TP |  |
| FP |  |
| TN |  |
| FN |  |
| More information | |
| Author contact required? (YES/NO) |  |
| Notes |  |

**Supplementary Table 3 Details of excluded studies**

| Excluded after title/abstract screened roughly（n=381） | |
| --- | --- |
| Causes | Records |
| Irrelevant topics | n=208 |
| Introductions or literature reviews of LI-RADS | n=114 |
| Machine-learning models or other models | n=10 |
| Systematic reviews and meta-analyses | n=8 |
| Protocols | n=3 |
| Case reports | n=7 |
| US LI-RADS | n=10 |
| CT/ MRI LI-RADS Treatment Response Algorithm | n=9 |
| Erratum or Letters or comments or conference catalogues | n=12 |
| Excluded after title/abstract screened meticulously (n=97) | |
| Causes | Records |
| Study patients didn’t meet high-risk criteria | n=3 |
| Either HCC or non-HCC malignancies were not included | n=30 |
| A minimum requirement of index text was not be meet | n=4 |
| Focus on inter-reader agreements without definite LI-RADS categories | n=7 |
| Focus on features without definite LI-RADS categories | n=29 |
| Irrelevant topics | n=15 |
| Abstracts with insufficient data | n=9 |
| **Excluded after full-text screened（n=21）** | |
| Cochrane studies still on-going（n=1） | |
| 1. NCT. Comparing SonoVue With Sonazoid Using CEUS-LIRADS in HCC. In:Comparing SonoVue With Sonazoid in the Performance of Contrast Enhanced Ultrasound The Liver Imaging Reporting and Data System (CEUS-LIRADS) in Diagosing HCC: a Prospective Blind Study, 2019. | |
| Number of HCC was contradictory in the context（n=1） | |
| 2. Ko A, Park HJ, Lee ES, et al. Comparison of the diagnostic performance of the 2017 and 2018 versions of LI-RADS for hepatocellular carcinoma on gadoxetic acid enhanced MRI. Clin Radiol 2020; 75:311-319. | |
| Patients don’t meet high-risk criteria（n=4） | |
| 3. Horvat N, Nikolovski I, Long N, et al. Imaging features of hepatocellular carcinoma compared to intrahepatic cholangiocarcinoma and combined tumor on MRI using liver imaging and data system (LI-RADS) version 2014. Abdominal radiology (New York) 2018; 43:169-178.  4. Laroia ST, Yadav K, Rastogi A, Kumar G, Kumar S, Sarin SK. Diagnostic efficacy of dynamic liver imaging using qualitative diagnostic algorithm versus LI-RADS v2018 lexicon for atypical versus classical HCC lesions: A decade of experience from a tertiary liver institute. European journal of radiology open 2020; 7:100219.  5. Lewis S, Peti S, Hectors SJ, et al. Volumetric quantitative histogram analysis using diffusion-weighted magnetic resonance imaging to differentiate HCC from other primary liver cancers. Abdom Radiol 2019; 44:912-922.  6. Seo N, Kim M, Rhee H. Hepatic sarcomatoid carcinoma: magnetic resonance imaging evaluation by using the liver imaging reporting and data system. Eur Radiol 2019; 29:3761-3771. | |
| Either HCC or non-HCC malignancies were not included（n=7） | |
| 7. Basha MAA, Refaat R, Mohammad FF, et al. The utility of diffusion-weighted imaging in improving the sensitivity of LI-RADS classification of small hepatic observations suspected of malignancy. Abdominal radiology (New York) 2019; 44:1773-1784.  8. Chernyak V, Flusberg M, Berman J, et al. Liver Imaging Reporting and Data System Version 2018: Impact on Categorization and Hepatocellular Carcinoma Staging. Liver transplantation: official publication of the American Association for the Study of Liver Diseases and the International Liver Transplantation Society 2019; 25:1488-1502.  9. Esposito A, Buscarino V, Raciti D, et al. Characterization of liver nodules in patients with chronic liver disease by MRI: performance of the Liver Imaging Reporting and Data System (LI-RADS v.2018) scale and its comparison with the Likert scale. La Radiologia medica 2020; 125:15-23.  10. Rosiak G, Podgorska J, Rosiak E, Cieszanowski A. Comparison of LI-RADS v.2017 and ESGAR Guidelines Imaging Criteria in HCC Diagnosis Using MRI with Hepatobiliary Contrast Agents. Biomed Res Int 2018; 2018:7465126.  11. Tan Z, Teoh WC, Wong KM, Wansaicheong GK, Sandrasegaran K. Analysis of comparative performance of CEUS and CECT/MR LI-RADS classification: Can CEUS dichotomize LI-RADS indeterminate lesions on CT or MRI? Clin Imaging. 2020 Jun; 62:63-68.  12. Zhang Y, Zhu F, Xu X, et al. Liver Imaging Reporting and Data System: Substantial Discordance Between CT and MR for Imaging Classification of Hepatic Nodules. Acad Radiol 2016; 23:344-352.  13. Zhang Y, Zhu F, Xu X, et al. Classifying CT/MR findings in patients with suspicion of hepatocellular carcinoma: Comparison of liver imaging reporting and data system and criteria-free Likert scale reporting models. Journal of magnetic resonance imaging: JMRI 2016; 43:373-383. | |
| Studies with insufficient data（n=8） | |
| 14. An C, Rhee H, Han K, et al. Added value of smooth hypointense rim in the hepatobiliary phase of gadoxetic acid-enhanced MRI in identifying tumour capsule and diagnosing hepatocellular carcinoma. Eur Radiol 2017; 27:2610-2618.  15. Cerny M, Bergeron C, Billiard J, et al. LI-RADS for MR Imaging Diagnosis of Hepatocellular Carcinoma: Performance of Major and Ancillary Features. Radiology 2018; 288:118-128.  16. Hahn ME, Almahmoud H, Mamidipalli A, et al. Histologic outcomes of observations categorized as LI-RADS m in clinical radiology reports. Abdom Radiol 2018; 43:1856-1857.  17. Jiang H, Liu X, Chen J, et al. Man or machine? Prospective comparison of the version 2018 EASL, LI-RADS criteria and a radiomics model to diagnose hepatocellular carcinoma. Cancer imaging: the official publication of the International Cancer Imaging Society 2019; 19:84.  18. Nakao S, Tanabe M, Okada M, et al. Liver imaging reporting and data system (LI-RADS) v2018: comparison between computed tomography and gadoxetic acid-enhanced magnetic resonance imaging. Jpn J Radiol 2019; 37:651-659.  19. Ominde ST, Mutala TM. Multicentre study on dynamic contrast computed tomography findings of focal liver lesions with clinical and histological correlation. SA journal of radiology 2019; 23:1667.  20. Schellhaas B, Wildner D, Pfeifer L, et al. LI-RADS-CEUS - Proposal for a Contrast-Enhanced Ultrasound Algorithm for the Diagnosis of Hepatocellular Carcinoma in High-Risk Populations. Ultraschall in der Medizin (Stuttgart, Germany : 1980) 2016; 37:627-634.  21. Schellhaas B, Pfeifer L, Kielisch C, Goertz RS, Neurath MF, Strobel D. Interobserver Agreement for Contrast-Enhanced Ultrasound (CEUS)-Based Standardized Algorithms for the Diagnosis of Hepatocellular Carcinoma in High-Risk Patients. Ultraschall in der Medizin (Stuttgart, Germany: 1980) 2018; 39:667-674. | |
| **Excluded studies with duplicated patients（n=10）** | |
| 1. Abd Alkhalik Basha M, Abd El Aziz El Sammak D, El Sammak AA. Diagnostic efficacy of the Liver Imaging-Reporting and Data System (LI-RADS) with CT imaging in categorising small nodules (10-20 mm) detected in the cirrhotic liver at screening ultrasound. Clin Radiol 2017; 72:901.  2. An C, Park S, Chung YE, et al. Curative Resection of Single Primary Hepatic Malignancy: Liver Imaging Reporting and Data System Category LR-M Portends a Worse Prognosis. AJR. American journal of roentgenology 2017; 209:576-583.  3. Chen L, Ruan S, Liang J, et al. Differentiation of intrahepatic cholangiocarcinoma from hepatocellular carcinoma in high-risk patients: A predictive model using contrast-enhanced ultrasound. World J Gastroentero 2018; 24:3786-3798.  4. Choi SH, Byun JH, Kim SY, et al. Liver Imaging Reporting and Data System v2014 With Gadoxetate Disodium-Enhanced Magnetic Resonance Imaging: Validation of LI-RADS Category 4 and 5 Criteria. Invest Radiol 2016; 51:483-490.  5. Joo I, Lee JM, Lee SM, Lee JS, Park JY, Han JK. Diagnostic accuracy of liver imaging reporting and data system (LI-RADS) v2014 for intrahepatic mass-forming cholangiocarcinomas in patients with chronic liver disease on gadoxetic acid-enhanced MRI. Journal of magnetic resonance imaging: JMRI 2016; 44:1330-1338.  6. Min JH, Kim JM, Kim YK, et al. Prospective Intraindividual Comparison of Magnetic Resonance Imaging with Gadoxetic Acid and Extracellular Contrast for Diagnosis of Hepatocellular Carcinomas Using the Liver Imaging Reporting and Data System. Hepatology (Baltimore, Md.) 2018; 68:2254-2266.  7. Ren A, Du J, Yang D, et al. The role of ancillary features for diagnosing hepatocellular carcinoma on CT: based on the Liver Imaging Reporting and Data System version 2017 algorithm. Clin Radiol. 2020; 75: 478.e25-478.e35.  8. Terzi E, De Bonis L, Leoni S, et al. CEUS LI-RADS are effective in predicting the risk hepatocellular carcinoma of liver nodules. Digest Liver Dis 2017; 49: e22.  9. Wang J, Feng S, Xu J, et al. Usefulness of the Contrast-Enhanced Ultrasound Liver Imaging Reporting and Data System in Diagnosing Focal Liver Lesions by Inexperienced Radiologists. Journal of ultrasound in medicine: official journal of the American Institute of Ultrasound in Medicine 2020.  10. Zheng W, Zhou J, Zou X, Li F, Wang J, Han F. Contrast enhanced ultrasound liver imaging reporting and data system version 2016: Application of the diagnostic algorithm and feedback on 1,773 liver lesions. Ultrasound Med Biol 2017; 43: S41-S42. | |

**Supplementary Table 4** The results of meta-regression of the sensitivity and specificity of CEUS LR5 for diagnosing HCC and CEUS LRM for characterizing OM.

| Parameter | CEUS LR5 for diagnosing HCC | | | | CEUS LRM for characterizing OM | | | |  |
| --- | --- | --- | --- | --- | --- | --- | --- | --- | --- |
|  | Sensitivity(95%CI) | *P* | Specificity(95%CI) | *P* | Sensitivity(95%CI) | *P* | Specificity(95%CI) | *P* |  |
| Country |  | <0.01* |  | 0.55 |  | 0.86 |  | <0.01* |  |
| Asian | 0.74 (0.72, 0.76) |  | 0.93 (0.87, 0.99) |  | 0.86 (0.75, 0.98) |  | 0.84 (0.78, 0.89) |  |  |
| Non-Asian | 0.62 (0.58, 0.65) |  | 0.94 (0.86, 1.00) |  | 0.79 (0.59, 1.00) |  | 0.96 (0.94, 0.99) |  |  |
| Study design |  | 0.45 |  | 0.66 |  | 0.55 |  | <0.01* |  |
| Retrospective | 0.71 (0.66, 0.76) |  | 0.94 (0.89, 0.99) |  | 0.86 (0.76, 0.96) |  | 0.87 (0.81, 0.93) |  |  |
| Prospective | 0.61 (0.50, 0.72) |  | 0.91 (0.76, 1.00) |  | 0.78 (0.47, 1.00) |  | 0.97 (0.94, 1.00) |  |  |
| Average lesion size≤20mm | | 0.03* |  | 0.45 |  | 0.82 |  | 0.44 |  |
| Yes | 0.69 (0.59, 0.78) |  | 0.92 (0.83, 1.00) |  | 0.83 (0.62, 1.00) |  | 0.92 (0.84, 1.00) |  |  |
| No | 0.69 (0.63, 0.76) |  | 0.94 (0.88, 1.00) |  | 0.85 (0.73, 0.97) |  | 0.89 (0.82, 0.97) |  |  |
| OM%≥10% |  | 0.03* |  | 0.66 |  | 0.47 |  | <0.01* |  |
| Yes | 0.67 (0.56, 0.78) |  | 0.93 (0.82, 1.00) |  | 0.85 (0.69, 1.00) |  | 0.75 (0.60, 0.90) |  |  |
| No | 0.70 (0.64, 0.76) |  | 0.94 (0.88, 0.99) |  | 0.86 (0.76, 0.96) |  | 0.93 (0.90, 0.96) |  |  |
| Reference standard | | 0.06 |  | 0.03* |  | 0.31 |  | <0.01* |  |
| Pathology | 0.72 (0.64, 0.80) |  | 0.89 (0.80, 0.99) |  | 0.81 (0.64, 0.97) |  | 0.79 (0.67, 0.90) |  |  |
| Mixed | 0.67 (0.60, 0.74) |  | 0.96 (0.91, 1.00) |  | 0.84 (0.73, 0.96) |  | 0.94 (0.91, 0.97) |  |  |

Abbreviations: CEUS, Contrast-Enhanced Ultrasound. HCC, hepatocellular carcinoma. OM, other non-HCC malignancies. *, *P*<0.05.

**Supplementary Table 5** The results of meta-regression of the sensitivity and specificity of CT/MRI LR5 for diagnosing HCC and CT/MRI LRM for characterizing OM**.**

| Parameter | CT/MRI LR5 for diagnosing HCC | | | | CT/MRI LRM for characterizing OM | | | |
| --- | --- | --- | --- | --- | --- | --- | --- | --- |
|  | Sensitivity(95%CI) | *P* | Specificity(95%CI) | *P* | Sensitivity(95%CI) | *P* | Specificity(95%CI) | *P* |
| Country |  | 0.33 |  | <0.01* |  | 0.07 |  | 0.02* |
| Asia | 0.69 (0.64, 0.74) |  | 0.92 (0.90, 0.95) |  | 0.62 (0.55, 0.69) |  | 0.96 (0.94, 0.98) |  |
| Non-Asian | 0.60 (0.51, 0.68) |  | 0.94 (0.91,0.97) |  | 0.69 (0.55, 0.82) |  | 0.92 (0.87, 0.98) |  |
| Study design |  | 0.06 |  | <0.01* |  | 0.15 |  | 0.59 |
| Retrospective | 0.67 (0.62, 0.72) |  | 0.93 (0.90, 0.95) |  | 0.63 (0.56, 0.69) |  | 0.96 (0.94, 0.97) |  |
| Prospective | 0.65 (0.56, 0.75) |  | 0.94 (0.91, 0.98) |  | 0.74 (0.55, 0.94) |  | 0.88 (0.73, 1.00) |  |
| Average lesion size≤20mm | | <0.01* |  | <0.01* |  | 0.17 |  | <0.01* |
| Yes | 0.61 (0.52, 0.70) |  | 0.95 (0.92, 0.98) |  | 0.57 (0.43, 0.72) |  | 0.93 (0.88, 0.97) |  |
| No | 0.68 (0.63, 0.73) |  | 0.92 (0.89, 0.95) |  | 0.64 (0.57, 0.71) |  | 0.96 (0.95, 0.98) |  |
| OM%≥10% |  | 0.08 |  | <0.01* |  | 0.45 |  | <0.01* |
| Yes | 0.71 (0.64, 0.79) |  | 0.87 (0.82, 0.92) |  | 0.66 (0.58, 0.73) |  | 0.94 (0.91, 0.97) |  |
| No | 0.64 (0.59, 0.70) |  | 0.95 (0.93, 0.97) |  | 0.60 (0.49, 0.70) |  | 0.96 (0.95, 0.98) |  |
| Index test |  | 0.01* |  | <0.01* |  | 0.03* |  | <0.01* |
| CT/MRI | 0.61 (0.59, 0.76) |  | 0.91 (0.87, 0.96) |  | 0.68 (0.58, 0.78) |  | 0.94 (0.89, 0.98) |  |
| MRI | 0.66 (0.61, 0.72) |  | 0.94 (0.91, 0.96) |  | 0.61 (0.54, 0.69) |  | 0.96 (0.94, 0.98) |  |
| CT/MRI LI-RADS version 2017/2018 | | 0.05* |  | <0.01* |  | 0.12 |  | <0.01* |
| Yes | 0.67 (0.62, 0.72) |  | 0.93 (0.91, 0.96) |  | 0.63 (0.55, 0.70) |  | 0.95 (0.93, 0.97) |  |
| No | 0.65 (0.56, 0.74) |  | 0.92 (0.88, 0.96) |  | 0.65 (0.54, 0.76) |  | 0.96 (0.93, 0.99) |  |
| Reference standard | | 0.10 |  | <0.01* |  | 0.24 |  | <0.01* |
| Pathology | 0.73 (0.66, 0.79) |  | 0.84 (0.80, 0.87) |  | 0.65 (0.57, 0.72) |  | 0.93 (0.90, 0.96) |  |
| Mixed | 0.63 (0.58, 0.69) |  | 0.95 (0.94, 0.96) |  | 0.62 (0.52, 0.72) |  | 0.97 (0.95, 0.98) |  |

Abbreviations: CT, computed tomography. MRI, magnetic resonance imaging. HCC, hepatocellular carcinoma. OM, other non-HCC malignancies. *, *P*<0.05.

**Supplementary Table 6** The diagnostic performance of LR5 for diagnosing HCC and LRM for characterizing OM with CEUS, CT and MRI separately.

|  | LR5 for diagnosing HCC | | | | LRM for characterizing OM | | | |
| --- | --- | --- | --- | --- | --- | --- | --- | --- |
|  | Sensitivity  (95%CI) | Specificity  (95%CI) | AUC (95%CI) | DOR (95%CI) | Sensitivity  (95%CI) | Specificity  (95%CI) | AUC (95%CI) | DOR (95%CI) |
| CEUS | 69% (64%, 74%) | 93% (87%, 97%) | 0.82 (0.79, 0.85) | 31.83 (14.04, 72.15) | 84% (71%, 92%) | 90% (83%, 95%) | 0.94 (0.91, 0.96) | 50.59 (22.16, 115.52) |
| CT | 64% (49%, 76%) | 92% (80%, 97%) | 0.85 (0.81, 0.88) | 20.10 (11.43, 35.35) | - | - | - | - |
| MRI | 68% (63%, 72%) | 93% (90%, 95%) | 0.88 (0.84, 0.90) | 28.38 (20.94, 38.45) | 63% (54%, 71%) | 96% (94%, 97%) | 0.92 (0.89, 0.94) | 36.97 (23.66, 57.76) |
| *p* value for DOR | | | | *p _CEUS vs CT_* = 0.54;  *p _CEUS vs MRI_* = 0.65;  *p _CT vs MRI_* = 0.44 |  | | | *p _CEUS vs MRI_* = 0.79 |

Abbreviations: CT, computed tomography. MRI, magnetic resonance imaging. HCC, hepatocellular carcinoma. OM, other non-HCC malignancies. AUC, area under the summary receiver operating characteristic curve. DOR, diagnostic odds ratios.
