# Supplementary figures and images for: Clinical Application of Liver Imaging Reporting and Data System for Characterizing Liver Neoplasms: A Meta-Analysis
Source: Diagnostics (Basel). 2021 Feb 17;11(2):323. doi: 10.3390/diagnostics11020323 (PMC7921912; doi:10.3390/diagnostics11020323)

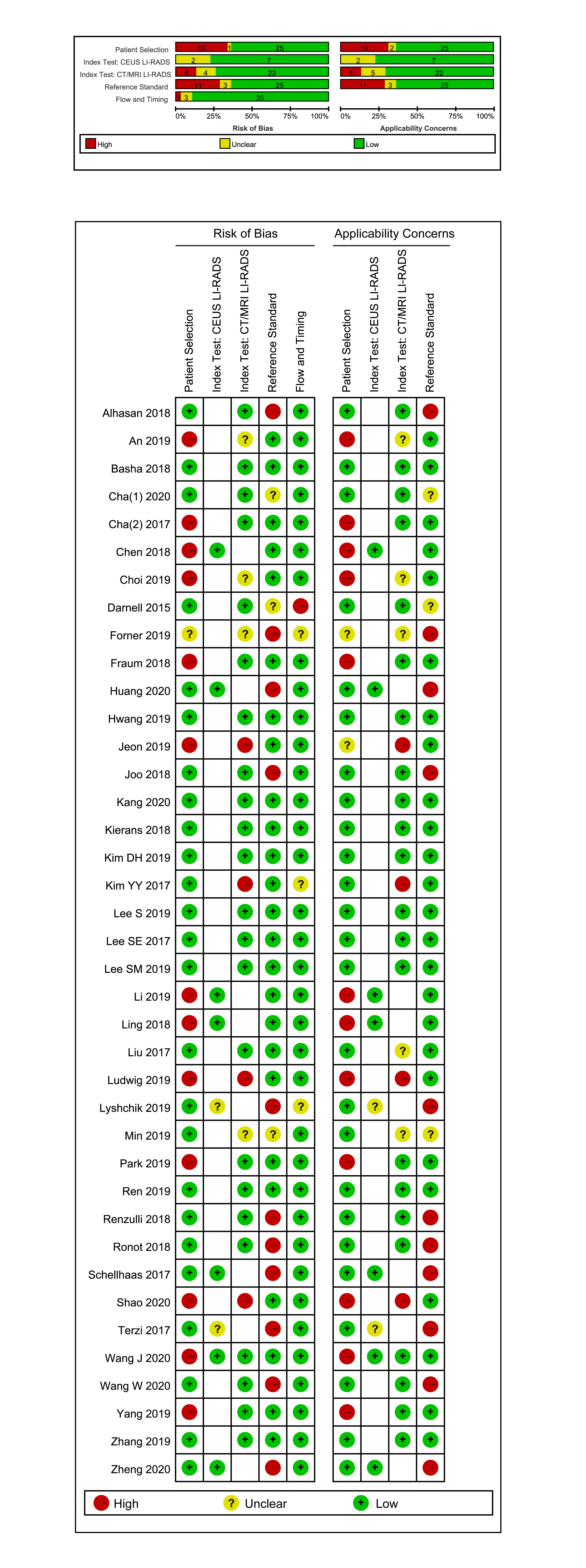

Supplement: Supplementary file 1 [file diagnostics-11-00323-s001.zip › supplementary file/Supplementary Figure 1.jpg]

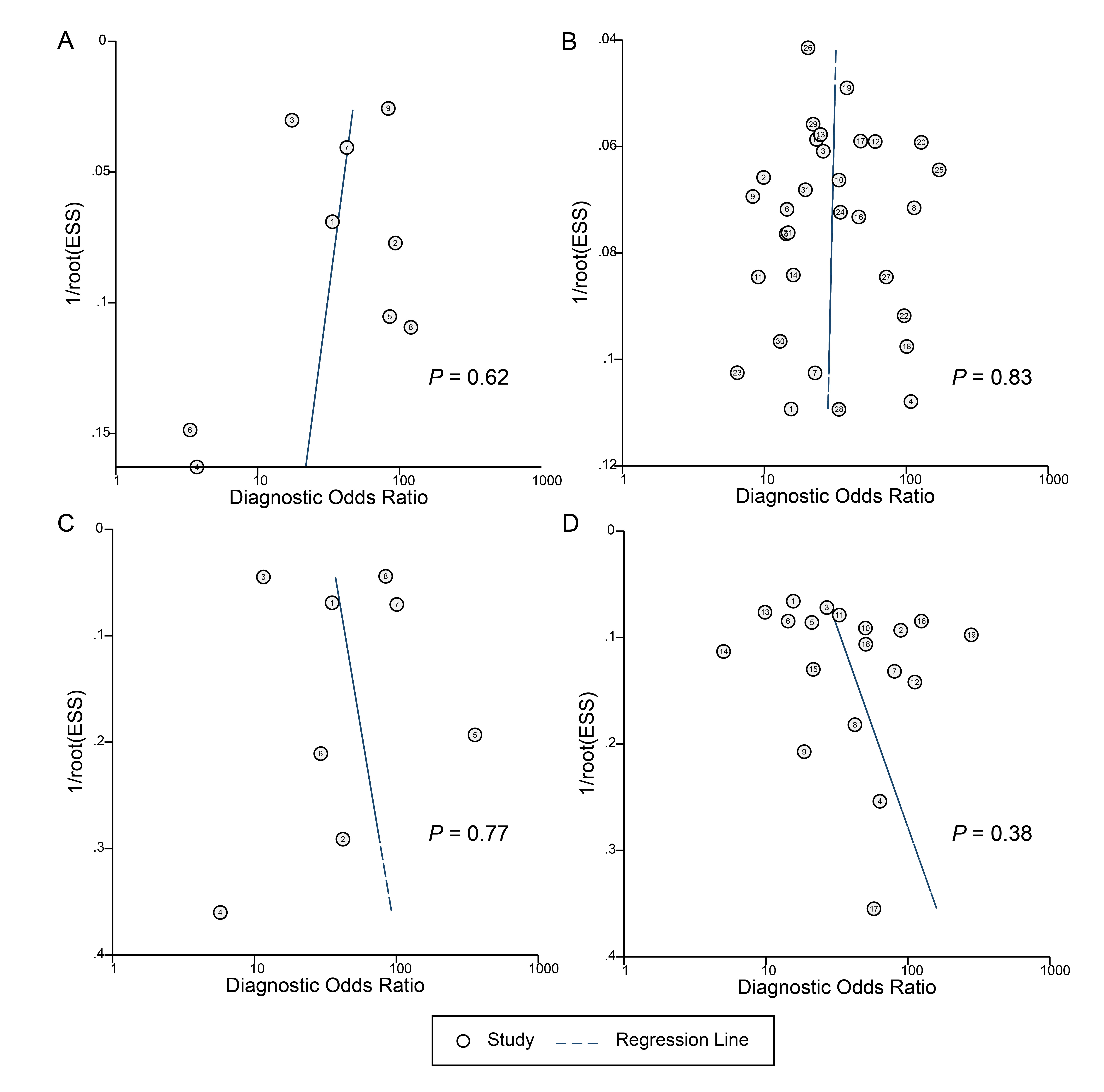

Supplement: Supplementary file 1 [file diagnostics-11-00323-s001.zip › supplementary file/Supplementary Figure 2.jpg]
